# Physicians’ perceptions on the impact of telemedicine on recruitment and retention in underserved areas: a descriptive study in Senegal

**DOI:** 10.1186/s12960-017-0242-z

**Published:** 2017-09-18

**Authors:** Birama Apho Ly, Ivy Lynn Bourgeault, Ronald Labonté, Mbayang Ndiaye Niang

**Affiliations:** 1 Population Health Program, University of Ottawa, Ottawa, Ontario Canada; 2 Public Health Teaching and Research Department, University of Sciences, Techniques and Technologies of Bamako, Bamako, BP: E 3624, Mali; 3 Telfer School of Management, University of Ottawa, Ottawa, Ontario Canada; 4 Faculty of Medicine, University of Ottawa, Ottawa, Ontario Canada; 5 Telemedicine Research and Expertise Interdisciplinary Centre, Faculty of Medicine, Cheikh Anta Diop University, Dakar, Senegal

**Keywords:** Physician perception, Telemedicine, Senegal, Recruitment and retention, Rural and remote areas, Underserved areas, Perception des médecins, Télémédecine, Sénégal, Recrutement et rétention, Régions rurales et éloignées, Régions desservies

## Abstract

**Background:**

Similar to many places, physicians in Senegal are unevenly distributed. Telemedicine is considered a potential solution to this problem. This study investigated the perceptions of Senegal’s physicians of the impact of telemedicine on their recruitment to and retention in underserved areas.

**Methods:**

We conducted individual interviews with a random sample of 60 physicians in Senegal, including 30 physicians working in public hospitals and 30 physicians working in district health centres between January and June 2014, as part of a mixed methods study. Data were collected using a semi-structured interview guide comprising both open- and close-ended questions. Interviews were recorded, transcribed and coded thematically using NVivo 10 software using a priori and emergent codes. Participants’ characteristics were analyzed descriptively using SPSS 23.

**Results:**

The impact of telemedicine on physicians’ recruitment and retention in underserved areas was perceived with some variability. Among the physicians who were interviewed, most (36) thought that telemedicine could have a positive impact on their recruitment and retention but many (24) believed the opposite. The advantages noted by the first included telemedicine’s ability to break their professional isolation and reduce the stress related to this, facilitate their distance learning and improve their working conditions. They did acknowledge that it is not sufficient in itself, an opinion also shared by physicians who did not believe that telemedicine could affect their recruitment and retention. Both identified contextual, economic, educational, family, individual, organizational and professional factors as influential.

**Conclusion:**

Based on these opinions of physicians, telemedicine promotion is one intervention that, alongside others, could be promoted to assist in addressing the multiple factors that influence physicians’ recruitment and retention in underserved areas.

## Résumé

**Introduction:**

Comme dans beaucoup de pays, les médecins sont très mal repartis au Sénégal. La télémédecine est considérée comme une solution efficace contre ce problème. Cette étude a exploré les perceptions des médecins du Sénégal sur l’impact de la télémédecine sur leur recrutement et leur rétention dans les régions desservies.

**Méthodes:**

Nous avons conduit des entretiens individuels auprès d’un échantillon aléatoire de 60 médecins du Sénégal dont 30 médecins des hôpitaux publics et 30 médecins des centres de santé de district entre janvier et juin 2014. Les données ont été collectées à travers un guide d’entretien semi-structuré comprenant des questions ouvertes et fermées. Les entretiens ont été enregistrés, transcrits et codés de manière thématique avec le logiciel NVivo 10. Les caractéristiques des participants ont été analysées de manière descriptive en utilisant le logiciel SPSS 23.

**Résultats:**

L’impact de la télémédecine sur le recrutement et la rétention des médecins dans les régions desservies était perçu différemment. La majorité des médecins interrogés (60%) ont estimé que la télémédecine pourrait avoir un impact positif sur leur recrutement et leur rétention, alors que beaucoup (40%) pensaient le contraire. Les avantages notés, par les premiers, incluaient la capacité de la télémédecine à briser leur isolement professionnel, faciliter leur formation à distance, améliorer leurs conditions de travail et réduire le stress lié à leur isolement professionnel. Ces médecins ont admis que la télémédecine n’est pas suffisante pour assurer leur recrutement et leur rétention. Cette opinion a été partagée par les médecins qui ont déclaré que la télémédecine ne pourrait pas avoir un impact positif sur leur recrutement et leur rétention. D’autres facteurs de recrutement et de rétention ont été identifiés à savoir les facteurs économiques, éducationnels, environnementaux, familiaux, individuels, organisationnels et professionnels.

**Conclusions:**

Ces résultats suggèrent que la télémédecine doit être associée à d’autres interventions pour agir sur les facteurs qui influencent le recrutement et la rétention des médecins dans les régions desservies.

## Background

In Senegal, physicians are more numerous (71%) in Dakar, the national capital, than in other regions [1]. This uneven distribution of physicians can jeopardize access to healthcare in underserved areas and create health inequities. To address this problem, several initiatives have been undertaken in Senegal in recent years including an increase in the supply of physicians through the creation of new medical schools and improvements in public sector management of physicians [2]. Despite these measures, the uneven distribution of physicians has worsened [2]. Telemedicine, defined as the practice of medicine at a distance [3], is a potential means to improve physicians’ recruitment to and retention in underserved areas [4, 5]. Several studies have demonstrated that it could have a positive impact on physicians’ recruitment and retention by allowing them to get advice from experts [6], reducing their professional isolation [7], and decreasing their feelings of work overload [8, 9]. Duplantie and colleagues, for example, demonstrated that it could have a positive impact on what they classified as educational, individual, organizational and professional factors, which also included an examination of contextual, economic and familial factors [5]. The educational factors identified by Duplantie and colleagues include exposure to practice in rural and remote regions during academic years, access to medical education and the opportunity to teach students. Individual factors include gender, age, being born and raised in a remote area, proximity of family and friends, and personal values. Organizational factors include quality of work conditions (access to resources, equipment and facilities) and the possibility of working in a team. Professional factors include opportunities for professional advancement, good relationship with patients, availability of support from the medical community and professional isolation [5]. All of these studies, however, are based in high-income countries. Senegal implemented both government and private initiatives of telemedicine, but little is known about telemedicine’s role in physicians’ recruitment and retention in underserved areas. No study of physicians’ perceptions of the role of telemedicine in their recruitment and retention in Senegal exists.

### Objective

The goals of this study were to determine the perceptions (both positive and negative) of Senegal’s physicians on the impact of telemedicine on their recruitment and retention in underserved areas and to identify the factors supporting their perceptions. The finality is to inform the potential strategies aiming to develop telemedicine in Senegal and to increase the number of physicians working in the underserved areas of this country.

## Methods

### Study design and participants

As part of a larger mixed methods study [10, 11], this research involved an exploratory qualitative approach entailing short individual interviews undertaken between January and June 2014. Participants comprised physicians who worked in Senegal’s public sector (public hospitals and district health centres). In 2014, there were 596 in public hospitals and 187 in district health centres. The Ministry of Health provided the list of these physicians from which a sample was derived.

### Sampling and recruitment

Although atypical in qualitative studies, we used random sampling to select 30 physicians working in each of the settings to reflect the multi-method nature of the larger study [10, 11]. These physicians were contacted to establish their willingness to participate in the study, which included both a quantitative survey and a qualitative interview component reported on here. Two physicians working in district health centres who were initially identified refused to participate and were replaced by two other physicians.

### Data collection

The 60 physicians were interviewed individually through face-to-face interviews by the lead author, and their responses recorded digitally for transcription. Interviews were guided by a brief semi-structured interview guide that included both open- and close-ended questions (Table 1) and lasted 5 to 15 min in length, typical of physician interviews. The interview guide was pilot-tested to ensure it was understandable and able to capture data of interest to our study in the short time period that our physician sample was willing to participate.

**Table 1:** Interview guide translated from the original version in French

Number	Questions
1	Could telemedicine have a positive impact on physicians’ recruitment to and retention in underserved areas?
2	If yes (or no), why (explain)?
3	Is telemedicine sufficient to recruit and retain physicians in underserved areas?
4	If yes (or no), why (explain)?
5	What other factors are likely to determine physicians’ recruitment and retention in underserved areas?

### Analysis

We began with a descriptive analysis of the demographic characteristics of participating physicians using SPSS 23. Then all the interview recordings were transcribed by the lead author and exported into NVivo 10. Using this qualitative analysis software, data were coded thematically (using both a priori and emergent codes) and the responses compared by demographic categories. This enabled an analysis of physicians’ characteristics according to their perception (positive or negative) of telemedicine’s impact on their recruitment to and retention in underserved areas. Subsequently, we described the other recruitment and retention factors mentioned by the participants according to the classification of Duplantie and colleagues which includes contextual, economic, educational, familial, individual, organizational and professional factors [5].

## Results

Table 2 presents the characteristics of the physicians who participated in our study.

**Table 2:** Physicians’ characteristics

Characteristics		Physicians working in public hospitals		Physicians working in district health centres	
		Number	Percent	Number	Percent
Age	≤ 30	2	6.7	0	0.0
	31–35	3	10.0	9	30.0
	36–40	3	10.0	9	30.0
	41–45	8	26.7	7	23.3
	45–50	6	20.0	4	13.3
	51–55	7	23.3	1	3.3
	56–60	0	0.0	0	0.0
	≥ 61	1	3.3	0	0.0
Sex	Male	25	83.3	28	93.3
	Female	5	16.7	2	6.7
Specialization	General practitioners	0	0.0	10	33.3
	Specialist physicians	30	100.0	20	66.7
Region	Dakar	24	80.0	2	6.7
	Outside Dakar	6	20.0	28	93.3
Total		30	100	30	100

The 30 physicians working in public hospitals were all specialist physicians and were mainly male except five women (16.7%). They were more numerous in Dakar (80%), and their average age was 44 years with a range of 29 to 61 years. The average age of the 30 physicians working in district health centres was 40 years with a range of 33 to 53 years. Physicians in district health centres were also predominantly male (93.3%) with only two female physicians (6.7%). Most district health centres physicians were also specialists (66.7%).

### Physicians’ perceptions

#### Positive perceptions

Of the 60 physicians who were interviewed, 36 (60%) thought that telemedicine can positively impact their recruitment to and retention in underserved areas, an opinion more prevalent among those working in public hospitals (22) than in district health centres (14). Of the 22 physicians who were working in public hospitals, 17 were in Dakar and five outside the city. Of the 14 physicians who were working in district health centres, more were general practitioners (10) than specialist physicians (4), with more working outside Dakar (12).

Of the 36 physicians with positive views, 16 participants thought that telemedicine would help diminish their professional isolation and allow them to communicate with their colleagues and experts. A male specialist physician working in a public hospital in Dakar, for example, said:

This is really important because if telemedicine is available, physicians who work outside Dakar will feel more connected to the other physicians who work in the bigger cities.

Another 16 participants thought that it would have a positive impact on their recruitment and their retention by facilitating distance learning, with a typical response being:

Of course, it could retain us because we don’t need to travel to Dakar to learn (Male specialist physician working in a district health centre outside Dakar)

Some 11 participants thought that it could improve their working conditions, which would encourage them to go and stay in underserved areas. Finally, four participants thought that it would help them to have the same level of information and knowledge as the physicians practicing in Dakar. As one participant summarized:

I think with telemedicine; information will circulate more quickly. We will then be informed at the same level as the physicians working in Dakar. (Male specialist physician working in a district health centre outside Dakar)

#### Negative perceptions

Of the 60 physicians who were interviewed, 24 thought that telemedicine would not have a positive impact on their recruitment to and retention in underserved areas. Most of those who held this opinion worked in district health centres (16) as opposed to public hospitals (8), of which most in this latter group (7) worked in Dakar. Of the 16 physicians who were working in district health centres, all were specialists and working outside Dakar.

According to these 24 physicians, telemedicine would not have a positive impact on their recruitment to and retention in underserved areas because it was not the only factor that determined physicians’ decisions. As one physician said:

You put all the possible and imaginable telemedicine machines, if their work environment is not good, if the prospects for professional advancement are not good, if the schools where they send their children are not good, if his wife cannot stay there, he will leave. (Male specialist physician working in a public hospital in Dakar)

This opinion was also shared by the 36 physicians who thought that telemedicine can have a positive impact on their recruitment to and retention in underserved areas. As one of the physicians noted:

To retain physicians in the regions, there is not just telemedicine. It is a factor, but it's not the only factor. There are other factors such as salary treatment, money, and all those things. People think that they are always monetarily well treated in Dakar than in the regions. (Male general practitioner working in a district health centre outside Dakar)

In the section below, we address the other issues which would need to be addressed alongside the availability of telemedicine.

### Other factors that determine physicians’ recruitment to and retention in underserved areas

Following the seven categories (contextual, economic, educational, familial, individual, organizational and professional) from the study of Duplantie and colleagues, participants’ opinions of the factors that would determine their recruitment to and retention in underserved areas are considered below.

#### Contextual factors

The contextual factors mentioned by the participants included poor living conditions in rural communities (12), bad climatic conditions (2) and limited number of patients (1). About the limited number of patients, one of the physicians said:

In some districts, there is nothing to do. The centre is in full Savannah. There are not a lot of patients. You get bored. (Male specialist physician working in a district health centre outside Dakar)

#### Economic factors

Economic factors included lack of incentives (22), lack of remoteness premium (11) and low income (2). For these physicians, there is no financial motivation to stay in underserved areas. One of the physicians said:

In Dakar, there is everything. This is not the case outside Dakar. Thus, discriminatory financial measures are needed. The physicians working outside Dakar should not have to be treated in the same way as the physicians working in Dakar. (Female specialist physician working in a district health centre outside Dakar)

#### Educational factors

For a number of participants (8), underserved areas offer fewer opportunities to train than Dakar. As one of the specialist physicians said:

If you are in Tambacounda, for example, you cannot participate in post-graduate studies. Thus, you cannot improve your knowledge. You work with only your knowledge acquired at school. Good things happen only in Dakar. *(*Male specialist physician working in a public hospital in Dakar)

#### Familial factors

Familial factors that were reported focused on the difficulties in finding schools for children (9) and the lack of job opportunities for spouses (*n* = 1). About the difficulties in finding schools for children, one physician commented:

The best investment is to invest in his children. We have to ensure their future. Competition is increasingly harder. Therefore, they have to go to the best schools. In remote regions, your children cannot go to school. (Male general practitioner working in a district health centre in Dakar)

Difficulties in finding schools for their children or job opportunities for their spouses led to their families staying in Dakar or in another place that offered more opportunities. This situation increased their family costs and pushed them to leave (or consider leaving) the regions that offered fewer opportunities for their families.

#### Individual factors

The only individual factor was mentioned by one of the physicians working in a district health centre outside of Dakar. He spoke of the desire to get closer to the region selected for retirement which, in his case, would be in Dakar.

Naturally, a physician just like other people needs to get closer to his family, to the region in which he wants to finish his career, in which he wants to spend the rest of his life. (Male specialist physician working in a district health centre outside Dakar)

#### Organizational factors

The organizational factors that were identified are poor working conditions (*n* = 14), extended stays in underserved areas (*n* = 8) and inequities in assigning physicians to their posts (*n* = 5). About the poor working conditions, one physician said:

This has nothing to do with telemedicine. They just want to have good working conditions. If you appoint a physician in a remote area in which there is nothing and ask him to do tasks normally reserved for nurses, he will leave. (Female specialist physician working in a public hospital in Dakar)

#### Professional factors

Professional factors highlighted by participants included lack of professional development (*n* = 2) and professional isolation (*n* = 3). The participants, who spoke to these professional factors, believed that Dakar offers more opportunities for professional development and fewer possibilities to be isolated professionally. One physician said:

After some years, we realize that we are silly. We realize that we are here, but Dakar offers more opportunities to develop professionally and more chance to stay in touch with experts. (Male specialist physician working in a public hospital outside Dakar)

These beliefs pushed them to stay in Dakar and jeopardized the chances to retain them in underserved areas.

## Discussion

These results show that the impact of telemedicine on physicians’ recruitment to and retention in underserved areas is perceived with some variability by Senegal’s physicians who worked in public hospitals and district health centres. Physicians who thought that telemedicine could have a positive impact on their recruitment to and retention in underserved areas emphasized how it could reduce stress related to their professional isolation and allow them to communicate with their colleagues and exchange information with experts, facilitate distance education, improve their working conditions and have the same level of information and knowledge as their colleagues working in Dakar. These results corroborate those of Brebner and colleagues, who found that telemedicine can have a positive impact on the recruitment of physicians in rural areas if it allows them to have experts’ opinion in the same way as their colleagues in larger cities [8]. They also corroborate those of other studies that found that telemedicine improved recruitment and retention by reducing professional isolation, allowing physicians to have the views of remote experts and reducing overload at work [7–9]. These results recall the advantages of telemedicine and invite Senegal’s policy-makers to consider them when promoting telemedicine. They also invite planners and researchers to consider them because they highlight the attributes of telemedicine that are valued by Senegal’s physicians and that can help to improve their recruitment and retention in underserved regions.

The physicians who thought that telemedicine was unlikely to have a positive impact on their recruitment to and retention in underserved areas believed that would be the case primarily because it was not the only factor that determined their decisions about where to locate, a finding consistent with many other studies [2, 5, 12]. Both groups of physicians (those positive and those negative about telemedicine’s role in recruitment and retention) identified a range of other important factors including contextual, economic, educational, familial, individual, organizational and professional factors.

The contextual factors that were mentioned by our participants are poor living conditions, bad climatic conditions and limited number of patients in underserved areas. On the poor living conditions, a healthy and less stressful environment is known to influence positively physicians’ recruitment [13]. It is also known that access to social and recreational activities can have a positive impact on both physicians’ recruitment and retention [14, 15]. Regarding the limited number of patients, this depends on the type and size of the population, which are recognized as factors that can positively impact physicians’ recruitment and retention [15, 16]. The small size of the population can lead to a small number of patients which can create boredom among physicians and aggravate their feelings of isolation. These results show the need to appoint physicians where they can find a satisfactory number of patients.

The identified economic factors inhibiting to underserved areas are lack of incentive, low income and lack of remoteness premium. It is well established that the remuneration of professionals has a positive influence on physicians’ recruitment [13, 15] and that payment of loans, benefits and compensations can encourage physicians’ recruitment and retention [17, 18]. These findings suggest that Senegal’s health authorities should introduce more incentive, income and remoteness premium for the physicians working in underserved areas.

Our participants also reported how educational factors such as training and access to continuing medical education can have a positive impact on physicians’ recruitment to and retention in underserved areas [19]. These results suggest that Senegal’s health authorities could provide more training opportunities to the physicians working in underserved areas, which could also become a telehealth extension to telemedicine services.

The identified familial factors were difficulties in finding schools for their children and lack of job opportunities for their spouses. These results are consistent with findings from several other studies [5, 18]. It also reflects the fact that, in Senegal, schooling and job opportunities are limited in underserved areas [2]. This situation leads physicians to live at a distance from their family because their children and spouses have to stay somewhere that offers more schooling and job opportunities. As one of our respondents noted, this situation is difficult socially and also economically because it leads to additional costs, a finding reported by others [2]. The physicians who live alone in underserved areas have to travel regularly to visit their family. They also need to rent two houses in two different regions if they do not have an official residence in their workplace [2]. These results show the need to improve schooling and job opportunities in underserved areas.

Regarding individual-level factors, only one of our participants mentioned the desire to get closer to the region selected for their retirement. There is little research exploring where physicians want to spend their retirement and what are the factors that motivate their choice. Similarly, there is not much research investigating the impact of this individual factor on physicians’ decision to leave or stay in an area. These results show the need to focus on these questions in future research.

Our results also revealed organizational factors such as poor working conditions, extended stays in underserved areas and inequities in assigning physicians to their post. These results corroborate those of Szafran and colleagues who found that good working conditions can encourage physicians’ recruitment [15]. They are also consistent with the results of the authors who believe that limited access to resources, equipment and infrastructure can prevent physicians’ retention in underserved regions [13, 20]. Finally, they are consistent with the results of Zurn and colleagues, whose studies of physicians in Senegal found that extended stays in underserved areas and the inequities in assigning physicians to their post are sometimes the most important factors that determine physicians’ recruitment and retention in underserved areas [2]. These results show the need to improve the working conditions of the physicians working in underserved areas, to enhance the management of physicians’ stay in their position and to apply equity when assigning physicians to their position.

Finally, the professional factors that were reported in this study are lack of professional development and professional isolation. Opportunities for professional development are well known to improve retention in underserved regions [15] while in Senegal, working in underserved areas is perceived to be a disadvantage for the rest of a professional career and a barrier to professional development [2]. Telemedicine could be useful in improving professional development opportunities in underserved areas and suggest a future policy direction for Senegal’s health authorities.

These findings show that physicians’ recruitment and retention in Senegal, as elsewhere, are influenced by many factors (contextual, economic, educational, family, individual, organizational and professional). Their implications for Senegal’s health authorities are self-evident. Indeed, they call for caution regarding the use of telemedicine in itself as a means to recruit and retain physicians in underserved areas.

### Limitations of the study

This study had a number of limitations reflecting sampling and data collection methods. First, the short length of the interviews, necessary to secure participation from some physicians, did not allow for in-depth analysis of influential factors. Interviews may also entail some social desirability bias. The participation in the study by so few female physicians did not enable an analysis of the influence of gender. This study also focused only on physicians working in public hospitals and district health centres. Telemedicine evolves very quickly, and our study reflects the perceptions of physicians given the telemedicine situation in Senegal in 2014.

## Conclusion

These results show that the majority of our respondents had a positive perception about the impact of telemedicine on their recruitment and retention in underserved areas. They also show that telemedicine is not the only factor that determines physicians’ decision to work and stay in underserved areas. They pinpoint other important factors that determine physicians’ recruitment and retention in underserved areas. Among these factors, there are contextual, economic, educational, familial, individual, organizational and professional factors. They suggest that telemedicine should be used with caution as a means to recruit and retain physicians in underserved areas. If it has to be used as such means, our results recommend its association with other measures to ensure better recruitment and retention of physicians in underserved areas. These measures may be equally, or even more, important than telemedicine in recruiting and retaining physicians. Knowing these results allows Senegal’s authorities to prioritize actions that are needed to improve physicians’ recruitment to and retention in underserved areas.
